# Opportunities and challenges related to ferroptosis in glioma and neuroblastoma

**DOI:** 10.3389/fonc.2023.1065994

**Published:** 2023-03-02

**Authors:** Huizhong Chi, Boyan Li, Qingtong Wang, Zijie Gao, Bowen Feng, Hao Xue, Gang Li

**Affiliations:** ^1^ Department of Neurosurgery, Qilu Hospital, Cheeloo College of Medicine and Institute of Brain and Brain-Inspired Science, Shandong University, Jinan, Shandong, China; ^2^ Shandong Key Laboratory of Brain Function Remodeling, Jinan, Shandong, China

**Keywords:** ferroptosis, neuroblastoma, glioblastoma, GPX4, immune

## Abstract

A newly identified form of cell death known as ferroptosis is characterized by the peroxidation of lipids in response to iron. Rapid progress in research on ferroptosis in glioma and neuroblastoma has promoted the exploitation of ferroptosis in related therapy. This manuscript provides a review of the findings on ferroptosis-related therapy in glioblastoma and neuroblastoma and outlines the mechanisms involved in ferroptosis in glioma and neuroblastoma. We summarize some recent data on traditional drugs, natural compounds and nanomedicines used as ferroptosis inducers in glioma and neuroblastoma, as well as some bioinformatic analyses of genes involved in ferroptosis. Moreover, we summarize some data on the associations of ferroptosis with the tumor immunotherapy and TMZ drug resistance. Finally, we discuss future directions for ferroptosis research in glioma and neuroblastoma and currently unresolved issues.

## Introduction

Despite their small percentage (approximately 1%) among all invasive cancer cases, malignant central nervous system (CNS) tumors are representative tumor types in children and adolescents as well as the major cause of death related to cancer in males younger than 40 and females younger than 20. As a result, malignant CNS tumors are the third and fourth leading cause of cancer-related death among individuals in the age ranges of 0-14 and over 40 years old, respectively (1, 2). Common malignant CNS tumors include glioma and neuroblastoma (NB). Gliomas account for 24.5% of all primary CNS tumors, while malignant tumors account for 80.9%. Gliomas usually have a poor prognosis. Glioblastoma (GBM) is a representative malignant CNS tumor (49.1% of all malignancies) with the shortest observed median patient survival. Although advanced therapeutic methods, including temozolomide (TMZ) therapy and tumor-treating fields (TTFields), are applied in the clinic, treated patients have a median survival time of only approximately 15 months. GBM has a poor prognosis, and only 5.8% of patients survive for five years (1–3). NB is another malignant tumor with a sympathetic nervous system origin and accounts for approximately 7-8% of childhood malignant tumor cases and approximately 15% of cancer-related deaths. Patients suffering from high-risk NB have a 5-year survival rate of less than 50% (4).

Ferroptosis is associated with iron and reactive oxygen species (ROS) and primarily results in cytological changes, including oxidative stress. As a result of strong membrane lipid peroxidation and oxidative stress, mitochondrial cristae are reduced or absent, the outer mitochondrial membrane is ruptured, and the mitochondrial membranes are condensed, resulting in weaker plasma membrane selective permeability and increased oxidative stress. At least three cytoprotective systems against ferroptosis with distinct subcellular localizations have been identified in recent studies: glutathione peroxidase 4 (GPX4) located in the cytoplasm and mitochondria; ferroptosis suppressor protein 1 (FSP1) located at the plasma membrane, which promotes ubiquinone regeneration; and dihydroorotate dehydrogenase (DHODH) located in the mitochondria. GPX4 can remarkably prevent ferroptosis by decreasing the levels of phospholipid hydroperoxides and thereby inhibiting lipid peroxidation mediated by lipoxygenase. FSP1, which promotes ubiquinone regeneration at the plasma membrane, uses NAD(P)H to catalyze the regeneration of nonmitochondrial coenzyme Q10 (CoQ10), which blocks ferroptosis by inhibiting lipid peroxide propagation. In parallel with mitochondrial GPX4, DHODH reduces ubiquinone (CoQ) to ubiquinol (CoQH2), an antioxidant capable of resisting ferroptotic activity, which inhibits ferroptosis within the inner mitochondrial membrane (independent of cytosolic GPX4 or FSP1) (5–7).

The prognosis of glioma and neuroblastoma is not particularly satisfactory. Currently, it is necessary to develop effective therapeutic approaches for glioma and neuroblastoma. A valid way to circumvent therapeutic resistance in cancer cells is targeting the ferroptotic pathway because of the high level of iron accumulation and the accompanying increase in ROS production. However, ferroptosis-related therapy application in glioma and neuroblastoma is still challenging because several aspects of the mechanisms of ferroptosis are still unclear. In this article, we present the progress in ferroptosis research in glioma and neuroblastoma and relevant future perspectives.

## Ferroptosis

Ferroptosis is a form of nonapoptotic cell death that results from the accumulation of intracellular iron and increased toxic lipid peroxide reactive oxygen species. In the prevention of ferroptosis, antioxidant systems can help decrease oxidative stress. Inhibition of an antioxidant system can contribute to the induction of ferroptosis in tumor cells. As a result, antioxidant systems are capable of remarkably regulating ferroptosis in cells and are also one of the major areas of research on ferroptosis at present.

The Xc system is also referred to as the cystine/glutamate reverse transporter protein. GPX4 essentially constitutes the selenoprotein family and mainly mediates the reduction of peroxides to the corresponding alcohol. This antioxidant system prevents ferroptosis by transporting cysteine through the Xc system for the synthesis of glutathione (GSH), which in turn helps GPX4 reduce peroxides. As a major antioxidant component, GSH participates in a wide range of redox reactions in the body to maintain physiological homeostasis. GPX4 can critically regulate ferroptosis and is known to determine cell fate. Upregulating or inhibiting these antioxidant systems to regulate ferroptosis can impact the development of various diseases. Moreover, studies have identified various drugs and molecules as inducers of ferroptosis that act by restricting Xc system activity (8, 9).

There is increasing evidence that inhibiting GPX4 activity does not necessarily lead to ferroptosis in cells. FSP1 on the plasma membrane reduces ubiquinone with NADPH as a cofactor, thereby preventing the peroxidation of lipids. GSH is not required as a cofactor for this process, nor does this process depend on GPX4. As a result, in contrast to GPX4, FSP1 may be regarded as a ferroptosis inhibitor, and the expression of FSP1 confirms the sensitivity of cells to ferroptosis (8, 10, 11).

DHODH, located in the inner mitochondrial membrane, is the enzyme involved in the 4^th^ rate-limiting step in pyrimidine biosynthesis and is capable of catalyzing dihydroorotic acid (DHO) to be oxidized to orotate (OA) and CoQ (ubiquitin) for further reduction to CoQH2 (ubiquinone), which is associated with the respiratory complex and affects electron transfer in the oxidative respiratory chain. Further studies have shown that inhibition of DHODH results in ferroptosis in cells with low GPX4 expression and increases the sensitivity of cells with high GPX4 expression to ferroptosis. DHODH can act synergistically with GPX4 to inhibit mitochondria-related ferroptosis without dependence on FSP1 (5, 12).

As one of the most important mechanisms regulating ferroptosis, antioxidant systems have always been important. With in-depth research, an increasing number of relevant molecules have been discovered, creating directions for further research and application of ferroptosis in glioma and neuroblastoma.

### Ferroptosis in glioma

**Figure 1:**
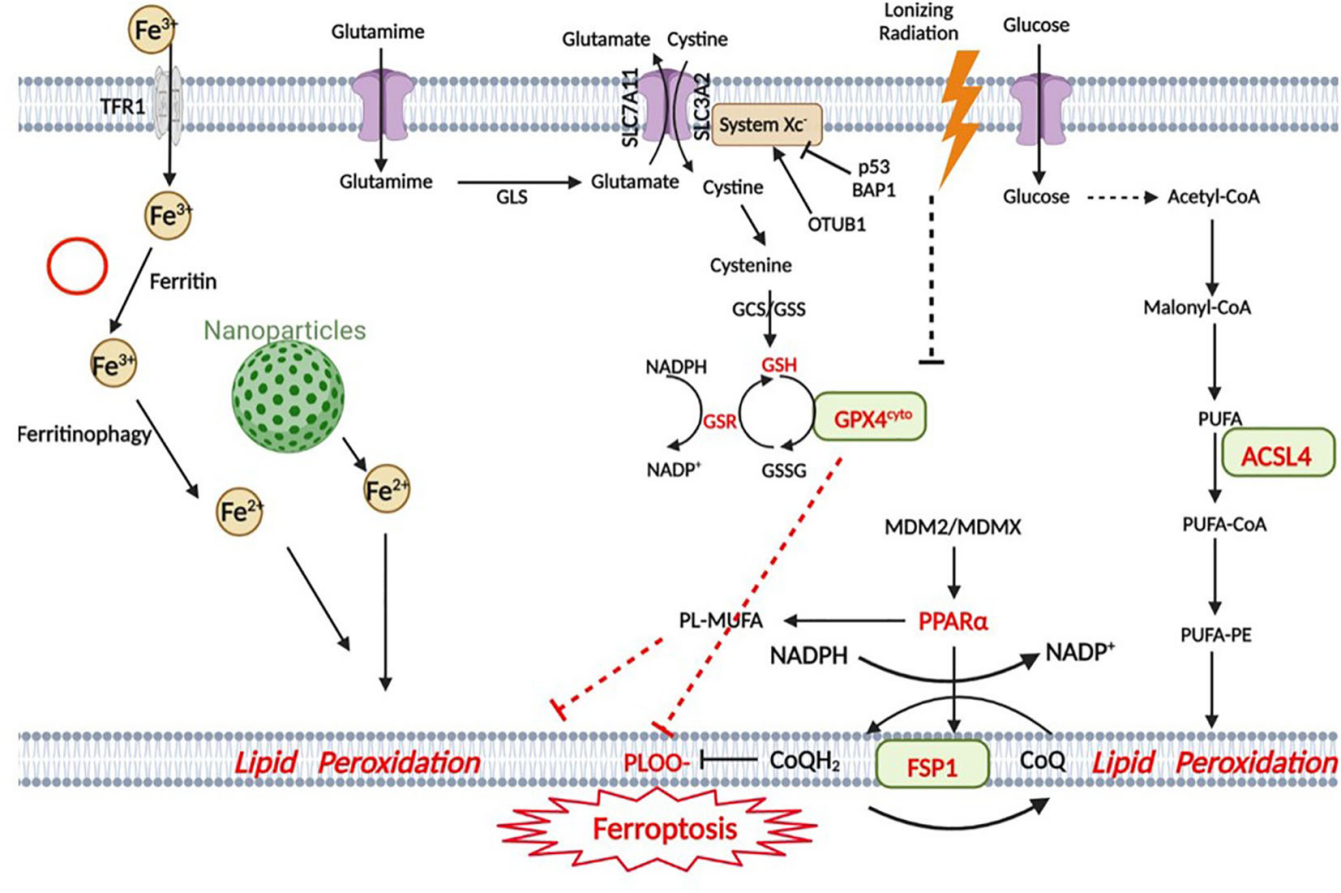
Snapshot of ferroptotic pathways. Ferroptosis in GBM is triggered by four main regulatory pathways: iron metabolism, the GPX4 pathway, the FSP1 pathway and lipid metabolism. In iron metabolism, Fe3+ is transported into the cell by TfR1 (transferrin receptor) and subsequently reduced to Fe2+, and some nanoparticles are involved in iron metabolism. The GPX4 pathway is the classic ferroptotic pathway, and the Xc- system plays an important regulatory role in this pathway. p53 is closely related to this pathway. The MDM2-MDMX complex regulates lipid metabolism by altering PPARα activity and ultimately interacts with the FSP1 protein. In lipid metabolism, AA (as well as other PUFAs) is metabolized by ACSL4 and eventually participates in lipid peroxidation. (Created with BioRender).

Glioma is a representative malignant CNS tumor. Currently, surgery, radiotherapy, chemotherapy, and tumor treatment fields (TTFields) are the most common treatments for clinical glioma, but they have a poor prognosis in patients, particularly those who suffer from high-grade gliomas, including GBM. The exploration of new therapeutic methods and therapeutic targets for glioma remains a hot spot. Targeting the ferroptotic pathway can serve as an effective treatment for glioma (**Figure 1**).

### Ferroptosis-related gene network in glioma

The Xc-GSH-GPX4 network serves as the primary antioxidant barrier against ferroptosis. As a direct target gene, recombinant solute carrier family 7, member 11 (SLC7A11) is repressed by p53. It is a key component of the cystine-glutamate antagonist system (xCT system), which mediates the uptake of extracellular cystine in exchange for glutamate within the cell. The direct interaction between ubiquitin hydrolase ovarian tumor domain protease domain, ubiquitin aldehyde binding protein 1 (OTUB1) and SLC7A11 stabilizes the SLC7A11 protein, and OTUB1 knockdown triggers SLC7A11 expression-dependent ferroptosis (13). Moreover, exogenous overexpression of NF-κB activating protein (NKAP) positively regulates SLC7A11 to promote cellular resistance to ferroptosis inducers (14).

Current research indicates that glutathione peroxidase 4 plays a critical role in ferroptosis. It has been demonstrated that a number of molecules affect the expression of GPX4 in gliomas to regulate ferroptosis. RSL3 (a GPX4 inhibitor) inactivates GPX4 and induces glycolytic dysfunction in glioma cells with reduced ATP and pyruvate content as well as HKII, PFKP, and PKM2 protein levels, which in turn induces ferroptosis (15). Knockdown of RNA-binding fragile X mental retardation syndrome-related protein 1 (FXR1) promotes TMZ-induced ferroptosis, thereby overcoming TMZ resistance. FXR1 has been proven to bind to the GPX4 mRNA transcript and exert a positive regulatory effect on GPX4 expression (16). γ-Glutamyltransferase 1 (GGT1) is an enzyme that cleaves extracellular glutathione. In GBM cells with GGT1 expression, drug inhibition or GGT1 deletion was shown to inhibit the increase in the intracellular glutathione levels induced by the cellular density and the cell viability affected by cystine deprivation. In addition, cystine deprivation led to glutathione depletion and ferroptosis in GBM cells deficient in GGT1 independent of a high cellular density. Exogenous expression of GGT1 in GBM cells deficient in GGT1 suppressed glutathione depletion and ferroptosis induced by cystine deprivation at a high density (17). Even more exciting, GPX4 expression is obviously reduced during tumor recurrence, whereas acyl-CoA synthetase long chain family member 4 (ACSL4) expression exhibits an obvious increase. Moreover, aldehyde dehydrogenase family 1, subfamily A3 (ALDH1A3) and FSP1 expression levels are also increased during recurrence, with the increase in ALDH1A3 expression being significant. It appears that exploiting the ferroptotic process may be a new therapeutic option, especially in patients with recurrent GBM (18). These findings provide new insights into the treatment of recurrent GBM and may contribute to the development of a basis for treating gliomas by targeting ferroptosis in an effective manner.

TP53 encodes p53 promoting cell cycle arrest, senescence, and apoptosis, which are three canonical functions of p53 involved in tumor suppression. This gene is the most frequently mutated tumor suppressor gene in all human cancers. The TP53 gene has been found to be activated under various conditions and to play an important role in the control of ferritin by regulating lipid, energy, and iron metabolism (19, 20). SLC7A11 is a key inhibitor of ferroptosis enhanced by p53. P62 (a stress-induced adaptor protein) inhibits ubiquitination, promotes ferroylation, and suppresses the expression of SLC7A11 in p53-mutant (MT) GBM, whereas it weakens ferroylation and increases SLC7A11 expression in p53-wild-type (WT) GBM (21). There is evidence that Rho family GTPase 1 (RND1) interacts with p53, leading to the deubiquitination of p53. In addition, overexpression of RND1 promotes the activity of the p53-SLC7A11 signaling pathway and triggers lipid peroxidation and siderosis in GBM cells (22). Reduced cystine uptake inhibits downstream GSH biosynthesis, impairing the ability of GPX4 to inhibit siderosis. In addition to downregulating SLC7A11 and impairing GSH biogenesis, p53 promotes ferroptosis through the regulation of other metabolic pathways. The rate-limiting enzyme in polyamine breakdown is arginine/arginine N1-acetyltransferase 1 (SAT1). In recent studies, we found that p53 could induce SAT1 expression, slowing the growth of xenograft tumors. As a result of SAT1 induction, arachidonate 15-lipoxygenase (ALOX15) was upregulated. The p53/SAT1/ALOX15 axis is therefore partially responsible for p53-mediated ferroptosis and tumor suppression (19, 23, 24). In addition, arachidonate 12-lipoxygenase (ALOX12) plays an important role in these functions. p53 promotes the activity of ALOX12. ALOX12 is bound by SLC7A11 and thus sequestered from its substrate, polyunsaturated fatty acids (PUFAs), including those esterified in membranes. ALOX12 is released when p53 downregulates SLC7A11, oxidizing membrane PUFAs and initiating ferroptosis (25, 26). Therefore, the p53/SLC7A11/ALOX12 axis is independent of the decrease in GSH biogenesis and GPX4 activity and is therefore a separate pathway from the p53/SLC7A11/GPX4 pathway. p53 inhibits the expression of SLC7A11 in the antiferroptosis system, and it can also inhibit the serine synthesis pathway as well as the transsulfuration pathway by inhibiting phosphoglycerate dehydrogenase and cystine synthase (CBS), respectively, thus limiting the expression of GSH (19, 27). Mouse double minute 2 homolog (MDM2) is the major E3 ubiquitin-protein ligase that degrades p53, but it is also a p53 target gene. MDM2 and its homolog MDMX can negatively regulate the tumor suppressor p53. Inhibition of MDM2 and MDMX leads to an increased FSP1 protein level, which in turn increases the coenzyme Q10 level. In addition, the MDM2-MDMX complex can alter peroxisome proliferator-activated receptor α (PPARα) activity to regulate lipid metabolism (28). In summary, several studies have been conducted on p53 and ferroptosis to date, and most support a role for p53 in ferroptosis (19, 29). Ferroptosis is promoted by the multiple roles of p53 in regulating cellular metabolism, particularly lipid, iron, ROS, and amino acid metabolism. It remains to be seen whether other metabolic target genes of p53 or metabolic processes modulated by p53 (including autophagy) contribute to p53’s ferroptosis-regulating role.

In recent years, ACSL4 was found to partially activate long-chain fatty acid metabolism and immune signal transduction, indicating that it might be a regulator of ferroptosis (30). ACSL4 overexpression was found to decrease GPX4 overexpression and increase ferroptosis marker levels, such as 5-hydroxyeicosatetraene (5-HETE), 12-HETE and 15-HETE, in glioma cells (31). miR-670-3p inhibits ferroptosis in glioblastoma cells by inhibiting ACSL4. As a result, inhibition of miR-670-3p could be an alternative strategy for the treatment of glioblastoma (32). Heat shock protein 90 (Hsp90) and dynamin-related protein 1 (Drp1) actively regulate and stabilize ACSL4 expression during ferroptosis in glioma triggered by erastin. Hsp90 overexpression and Drp1 dephosphorylation change the mitochondrial morphology and increase lipid peroxidation mediated by ACSL4 to promote ferroptosis (33).

GPX7 is another member of the glutathione peroxidase family (GPX) and participates in oxidative stress and tumorigenesis. GPX7 silencing enhances oxidative stress associated with ferroptosis in glioma cells, while GXP7 deletion sensitizes gliomas to ferroptosis induced by erastin. In addition, miR-29b was found to repress GPX7 expression directly after transcription (34).

Recent studies have confirmed that tetrahydrobiopterin (BH4), a significant cofactor for multiple enzymes, can remarkably inhibit ferroptosis. The GTP cyclohydrolase 1 (GCH1)-BH4 axis controls BH4 synthesis and reduces intracellular CoQ and ROS accumulation, thereby leading to ferroptosis inhibition. In addition, GCH1/BH4 exerts a selective inhibitory impact on nuclear receptor coactivator 4 (NCOA4)-mediated ferritin autophagy and affects iron metabolism (8, 35). This provides a new direction for ferroptosis research in glioma. Coatomer protein complex subunit zeta 1 (COPZ1) negatively regulates NCOA4 activity, and COPZ1 knockdown induces NCOA4-mediated ferritin phagocytosis (36). Downregulation of matrix-remodeling-associated protein 8 (MXRA8) increases the intracellular levels of lipid peroxidation in glioma cells, leads to NCOA4 upregulation and inhibits ferritin heavy chain 1 (FTH1). MXRA8 is significantly associated with various infiltrating immune cells, such as NK cells, macrophages, and neutrophils. MXRA8 knockdown in glioma cells attenuates M2 macrophage infiltration. Accordingly, MXRA8 facilitates glioma progression and critically affects glioma ferroptosis and the immune microenvironment (37).

The transcription factor nuclear factor erythroid 2-related factor 2 (Nrf2) controls the expression of genes associated with oxidative stress and can reliably maintain redox stability and resistance to oxidative stress. High levels of NRF2 lead to sensitivity in glioblastoma dependent on the expression of its proferroptotic target ATP binding cassette subfamily C member 1 (ABCC1), resulting in GSH depletion upon blockade of the Xc system by erastin (38).

With ongoing research progress, the mechanisms regulating ferroptosis are becoming increasingly clear. Further research on the ferroptosis-related gene network will provide new ideas and broad opportunities for the treatment of glioma, not only primary high-grade gliomas such as GBM but also recurrent gliomas. However, there are a few issues that require further exploration. For example, we must determine how to more effectively and precisely induce ferroptosis in glioma cells and improve the efficacy and safety of this treatment.

### Ferroptosis-related compounds in glioma

Chemotherapy is one of the basic therapeutic strategies for glioma. TMZ is currently one of the first-line chemotherapeutic drugs for glioma, especially high-grade glioma. However, with the widespread use of TMZ, the median survival time of GBM patients has improved by only approximately 2.6 months. Frustratingly, as GBM patients receive long-term TMZ therapy, resistance inevitably develops, resulting in treatment efficacy dropping significantly or even disappearing. New replacement drug regimens remain to be developed (39). In the **Table 1**, we list the recent advances in drug-induced glioma ferroptosis for the treatment of glioma (40–51).

**Table 1:** Ferroptosis-inducing drugs in GBM.

Drug name	Target	Cell line and Animals	Pathway	Impact on Ferroptosis	Ref.
Dihydroartemisinin (DHA)	GPX4	U251, U373 and HT22	PERK/ATF4/HSPA5 pathway	• Increase GPX4 expression and activity • Upregulate ATF4	(40)
Brucine	ATF3	U118, U87, U251 and A172	Trigger ATF3 upregulation and translocation into the nucleus through activation of ER stress	• Promote H2O2 accumulation through upregulation of NOX4 and SOD1 • Downregulate catalase and xCT	(41)
Pseudolaric acid B (PAB)	Transferrin receptor	Rat C6 and human SHG-44, U87 and U251 glioma cells	Upregulate transferrin receptor; p53-mediated xCT pathway	• Upregulate transferrin receptor • Promote H2O2 and lipid peroxide generation • Deplete intracellular GSH *via* the xCT pathway mediated by p53	(42)
Amentoflavone (AF)	Autophagy-dependent ferroptosis	U251 and U373 glioma cells	AMPK/mTOR pathway	• Decrease the GSH level in tumor tissue • Increase the expression of LC3B, Beclin1, ATG5, and ATG7	(43)
RSL3	GPX4	U87 and U251	NF-κB pathway	• Increase the concentration of lipid ROS and downregulate proteins related to ferroptosis (GPX4, ATF4, and SLC7A11) • Activate the NF-κB pathway	(44)
Dihydrotanshinone I	GPX4 and ACSL4	U87 and U251	GPX4 and ACSL4 pathway	• Decrease the GPX4 level and increase the ACSL4 level • Reduce the GSH/GSSG ratio	(45)
Apatinib	Nrf2	U87 and U251	VEGFR2/Nrf2/Keap1 pathway	• Decrease Nrf2 and p-VEGFR2 expression	(46)
Sevoflurane	GPX4 and ATF4	U87 and U251	ATF4-CHAC1 pathway	• Increase ROS levels and the Fe^2+^ concentration • Downregulate GPX4, upregulate transferrin and activate ATF4	(47)
Plumbagin	xCT and GPX4	U87, U251, C6 and GL261	NQO1/GPX4 pathway	• Downregulate xCT and GPX4 • Increase NQO1 activity	(48)
Curcumin analog (ALZ003)	FBXL2	U87 and A172	GPX4 pathway	• Decrease GPX4 expression • Induce lipid peroxidation and ROS accumulation	(49)
Capsaicin	ACSL4 and GPX4	U87 and U251	GPX4 and ACSL4 pathway	• Increase ACSL4, 5-HETE, MDA and TOS levels and decrease GPX4, GSH and TAS levels	(50)
Boric acid (BA)	ACSL4 and GPX4	GBM C6 cells	ACSL4/GPx4SEMA3F/NP2 pathways	• Increase ACSL4 levels and decrease GPX4 levels • Upregulate SEMA3F/NP2	(51)

From the **Table 1**, we can see that many drugs used in the past also have a good effect on ferroptosis and that they inhibit the growth of glioma cells by targeting different ferroptotic pathways and target genes. This suggests that it is possible to find new uses for these drugs related to treatment targeting ferroptosis.

### Ferroptosis-inducing nanoparticles

The use of rationally designed nanomaterials for the treatment of cancer is an emerging field that has led to tremendous medical success. The administration of ferroptosis-inducing nanoformulations with accurately tuned physicochemical properties is as an extended and feasible therapeutic strategy for tumors. We compiled recent research advances related to the induction of ferroptosis in glioma cells by nanomaterials. (**Table 2**) (52–56).

**Table 2:** Ferroptosis-inducing nanoparticles in GBM.

Nanoparticle name	Target	Cell line and Animals	Impact on Ferroptosis	Ref.
(FA)/Pt-si-GPX4@IONPs	GPX4	U87MG, P3#GBM and NHA	• Increase iron (Fe^2+^ and Fe^3+^) levels; increase H2O2 levels through the activation of lower NOX • Inhibit GPX4 expression	(52)
PIOC@CM NPs	GPX4	C6	• Increase the ROS level and deplete GSH upon ultrasonic irradiation • Inhibit GPX4 expression	(53)
Fe3O4-siPD-L1@M-BV2	GPX4 and PD-L1	GL261, HT-22 and BV2	• Induce the maturation of DCs and decrease the protein expression of PD-L1 • Inhibit GPX4 expression	(54)
cRGD/Pt + DOX@GFNPs (RPDGs)	N/A	U87 and NHA	• Deplete GSH and elevate the ROS level	(55)
Fe3O4@mSiO2 NPs	DHODH and GPX4	LN229 and A172	• Inhibit GPX4 and DHODH expression • Deplete GSH and elevate the ROS level	(56)

These different nanodrugs offer a new direction for ferroptosis-based therapy for gliomas. The different designs are very interesting. It is beneficial to generate nanoparticles encapsulated with Fe3O4 and Ce6 acoustic sensitizers, and external loading of C6 cell membranes is performed to achieve tumor cell enrichment of the material. Transient opening of the blood−brain barrier can be achieved with focused ultrasound (US). This sonodynamic therapy (SDT) combines targeting of ferroptosis in glioma cells with SDT (53). However, noninvasive destruction of the blood−brain barrier (BBB) by focused ultrasound may lead to the entry and/or exit of some harmful substances at the same time. In addition, the combination of ferroptosis-targeting therapy and immunotherapy is also a good treatment strategy (54). A membrane-modified drug delivery system was constructed by loading small interfering RNA targeting programmed cell death 1 ligand 1 (PD-L1) on Fe3O4 and externally on the BV2 cell membrane. This system promoted synergy between ferroptosis induction and immunotherapy by reducing the expression of PD-L1 *in situ* in drug-resistant GBM tissues, which was combined with the effect of ferroptosis induction by Fe2+ in Fe3O4. Some studies have also been conducted on the combination of chemotherapeutic drugs with nanomaterials (55). Gallic iron nanoparticles combined with the chemotherapeutic agent cisplatin produce a dual killing effect. The material’s photothermal responsiveness and ability to be imaged by MRI provide a new way to treat GBM. Recent studies have also combined exosomes with nanomaterials to create a composite ferroptosis platform (56). A study engineered exosomes by modifying the ANG-targeting peptide on the surface of the exosomes, giving them a greater ability to cross the blood−brain barrier. Next, they constructed a nanomaterial with an Fe3O4 core, a mesoporous silicon shell and a modified anti-CD63 antibody on the surface of the mesoporous silicon shell for branching exosomes. Ultimately, the ferroptosis-related therapeutic effect of the system was achieved by encapsulating a drug or small interfering RNA targeting a critical ferroptotic pathway in the mesoporous silicon shell and exosomes.

In general, the different designs are interesting and well designed. In conclusion, to achieve ferroptosis-targeted therapy with nanomaterials, the following steps must be achieved: blood−brain barrier penetration, tumor targeting, and ferroptosis induction. Nanomaterials with properties that enable these events may be new agents for glioma therapy in the future. However, the design of different nanomaterials is relatively complicated, such as the camouflage achieved with different cell membranes and the encapsulation of different drugs, and further improvements and validation in industrial production and human experimental safety are still needed. However, we believe that with the continuous progress of medical-industrial crossover technology, an increasing number of nanoagents will start to capture attention and provide new insights for the treatment of glioma in combination with ferroptosis-inducing agents.

### Ferroptosis and TMZ resistance

TMZ is still a most effective drugs for glioma chemotherapy. Ferroptosis can considerably affect TMZ resistance in glioma, and ferroptosis resistance may serve as a mechanism of TMZ resistance in glioma. TMZ increases LDH, MDA and iron levels and decreases GSH levels in glioma cells to induce ferroptosis. In addition, ROS levels and DMT1 expression are elevated, and GPX4 expression is decreased in cells treated with temozolomide; these events are under the regulation of the Nrf2/HO-1 pathway (57).

In addition to ferroptosis inducers and xCT inhibitors, quinacrine (a compound capable of crossing the blood−brain barrier) has been found to impair autophagy but increase the sensitivity of glioblastoma stem cells (GSCs) to TMZ and trigger ferroptosis in GSCs (58). A long non-coding RNA LINC01564 promotes glioma cell resistance to TMZ by upregulating Nrf2 expression, which counteracts the effects of MAPK8 ablation on glioma cell apoptosis and ferroptosis to inhibit ferroptosis (59).

Further study of the ferroptosis mechanism in glioma TMZ resistance will contribute to new insights into the clinical reversal of glioma TMZ chemoresistance. However, details are still needed for clinical application.

### Ferroptosis and immunotherapy

One of the most effective ways to treat cancer is to induce tumor cell death. Immunotherapy is considered a milestone in precision medicine. It elicits significant therapeutic responses in patients who have developed resistance to other conventional therapies (60). However, immunotherapy is not particularly effective in glioma, especially in GBM. A growing body of research suggests that the glioma immunosuppressive microenvironment (GIME) contributes to the poor efficacy of glioma immunotherapy (61–63). The rapid proliferation of gliomas creates a harsh microenvironment that is acidic with nutrient scarcity and hypoxia (64–66). As a result, immune cells become immunosuppressive or inactive or die (67, 68), whereas glioma cells may be able to adapt to this harsh microenvironment due to their substantial plasticity (69, 70). Additionally, the blood−brain barrier prevents immune cells from migrating to tumors (71, 72). Furthermore, many suppressive cytokines secreted by gliomas (73) and suppressive immune cells suppress the antitumor activity of immune cells (74, 75). Furthermore, glioma cells can secrete a large number of cytokines to capture immune cells. Glioma cells are able to escape immune surveillance in this case (62, 76). To treat glioma successfully, it is therefore essential to remodel the immune microenvironment.

This is of great importance for improving traditional drug resistance, as ferroptosis is closely related to antitumor immunity and the immune microenvironment. Calreticulin (CRT), a soluble chaperone associated with the endoplasmic reticulum (ER), is one of the proteins that regulates the tumor microenvironment. As a result of ferroptosis, CRT is translocated onto tumor cells, where it can induce a robust immune response against the tumor (77). Neutrophils have been reported participate in apoptosis by accumulating iron-dependent lipid peroxide, which results in iron atrophy in GBMs. Intratumoral depletion of ACSL4 or overexpression of GPX4 reduces tumor necrosis and aggressiveness (78). By harnessing the cytotoxic potential of the immune system, notably that of tumor-specific cytotoxic T cells, immunotherapy is a promising strategy to treat malignancies. As a result of their antitumor effects, CD8+ T cells are a crucial component of the tumor microenvironment; they also play a key role during every stage of tumor development. Ferroptosis is a metabolic vulnerability of tumor-specific CD8+ T cells, whereas GPX4-deficient T cells display a high sensitivity to ferroptosis and are thus incapable of exerting antitumor effects. Overexpression of GPX4 inhibits ferroptosis in CD8+ T cells and simultaneously restores the production of cytotoxic cytokines *in vitro* or increases the number of tumor-infiltrating CD8+ T cells *in vivo*, thereby enhancing tumor control (79–81). In contrast, increased ferroptosis facilitates immune cell activation and infiltration but attenuates the killing of tumor cells through cytotoxic activity (82). Moreover, enhanced ferroptosis contributes to the recruitment of tumor-associated macrophages (TAMs) and M2 polarization (83). These factors contribute to the creation of an immunosuppressive immune microenvironment, which may lead to immune escape. Further studies are needed to balance the dual effects in the future.

Interestingly, ferroptosis exhibits immunogenicity *in vitro* and *in vivo*, triggering a vaccination-like effect in immunocompetent mice, in which ATP and high mobility group box 1 (HMGB1), the most typical injury-related molecular patterns associated with immunogenic cell death, can be passively released and act as immunogenic signals that affect the immunogenicity of early ferroptotic cancer cells (84). Thus, this novel discovery provides a new direction for vaccine therapy.

Clinical trials of immune checkpoint inhibitors (ICIs) have demonstrated a broad clinical impact and early success. Some but not all cases of ICI response have been associated with the expression of immune checkpoint molecules, including PD-1 ligand (PD-L1) (85). Some patients with PD-L1-positive tumors do not respond to treatment, while some patients with PD-L1-negative tumors may benefit from ICI therapy due to tumor heterogeneity (86, 87). TYRO3 inhibits anti-PD-1/PD-L1-induced ferrogenesis in tumor cells by suppressing the AKT/NRF2 axis and amplifies a favorable tumor microenvironment by reducing the ratio of M1/M2 macrophages, thus contributing to the efficacy of anti-PD-1/PD-L1 therapy (88). More effective immune checkpoints or more valid regulatory pathways need to be explored to overcome resistance in glioma patients.

Although ICI immunotherapy has been shown to have significant positive effects in some cancer patients, there is still evidence of drug resistance in many tumors, including GBM, due to tumor heterogeneity, low tumor-infiltrating T-cell (TIL) levels, loss of target antigens and off-target toxicity (89, 90). Chimeric antigen receptor T (CAR-T) cell immunotherapy targeting neoantigens that are derived from somatic mutations and expressed on only tumor cells has led to a new approach in cancer immunotherapy. CAR-T cell therapy has achieved certain success in both basic research and small-scale clinical research (91). B7-H3 (CD276) is expressed on CNS tumors, and B7-H3-specific CAR-T cells were designed for therapy in diffuse intrinsic pontine glioma (DIPG), producing exciting results (92). Frustratingly, there are no cases of relevant CAR-T cells designed to induce ferroptosis in gliomas. In addition, taking advantage of CAR-T cells to transform the immune microenvironment and enhance ferroptosis in tumor cells is a novel direction to be explored.

In conclusion, with increasing research, immunotherapy is becoming more specific and individualized, which provides opportunities for therapy in glioma. The effects of ferroptosis and immunotherapy are bidirectional, i.e., ferroptosis can further influence the effect of immunotherapy by affecting the immune microenvironment, and the effect of immunotherapy can be further enhanced by enhancing ferroptosis. However, there are still some details and limits that need to be further researched for glioma therapy.

### Potential Biomarkers of Ferroptosis

With the development of sequencing technology and the creation of databases, bioinformatic analysis now plays an important role in identifying potential targets and drug effects and predicting prognosis. We compiled the recently published literature on biogenic analysis to provide potential new ideas for future research (**Supplement 1**) (93–105). As shown in the table, different studies identified different targets, and some of the studies explored several targets.

Although a large number of bioinformatic analysis studies currently provide us with ferroptosis-related targets in low-grade glioma (LGG) and GBM, they still have many limitations and points of controversy due to the lack of rigorous experimental support. Many of the studies relied on only computer technology. Bioinformatic analysis may be a future direction, but the validity and clinical significance of the molecules identified with this approach need to be further explored.

### Ferroptosis in neuroblastoma

Neuroblastoma is one of the most prevalent extracranial tumors in children, accounting for the majority of childhood cancer-related deaths, especially in high-risk cases. High-risk NB is characterized by the appearance of this disease after the age of 18 months, the amplification of MYCN (MYCN Proto-Oncogene, BHLH Transcription Factor), or the activation of mechanisms for telomere maintenance (106, 107). The scientific community is committed to finding new strategies related to ferroptosis based on the characteristics of high-risk NB as a potential therapy for high-risk NB (108, 109).

### The ferroptosis-related gene network in neuroblastoma

The characteristics of NB are significantly different from those of glioma, and the focus is also different. Genomic amplification of the oncogene MYCN acts as an essential oncogenic event in high-risk NB, occurring in approximately 50% of high-risk cases, and MYCN amplification is strongly related to a poor NB prognosis (OS < 50%). MYCN-amplified NB shows a system-dependent increase in the level of the Xc-cystine/glutamate reverse transporter protein for ROS detoxification mediated by increased transcription of this receptor (108). As a result, MYCN amplification may be a potent target in NB, and much research has focused on this aspect. MYCN induces massive lipid peroxidation when consuming cysteine, the rate-limiting amino acid in the biosynthesis of GSH, which sensitizes cells to ferroptosis. When the uptake of cysteine in MYCN-amplified pediatric NB is restricted, the use of cysteine in protein synthesis can inevitably cause GSH-induced ferroptosis and spontaneous tumor regression of low-risk NB (110). In addition, NB cells with amplified MYCN can easily undergo ferroptosis due to the upregulation of TFRC-encoded transferrin receptor 1, which reprograms cellular iron metabolism through the upregulation of TFRC (Transferrin Receptor) expression. TFRC-encoded transferrin receptor 1 is a pivotal iron transporter protein on the cell membrane, and elevated iron uptake facilitates the accumulation of unstable iron pools, resulting in elevated lipid peroxide production. TFRC overexpression in NB cells is also capable of inducing selective sensitivity to ferroptosis inhibition by GPX4 (111, 112).

Ferroportin (Fpn) is the only iron export protein that partially regulates the intracellular iron concentration. Fpn knockdown has been shown to increase the accumulation of iron-dependent lipid ROS to accelerate erastin-induced ferroptosis, and Fpn may be an appropriate target for NB treatment (113). Mitochondrial ferritin (FtMt), a kind of iron storage protein in the mitochondria, also exerts a protective effect during erastin-induced ferroptosis (114). Recent mechanistic studies have shown that downregulation of CDC27 results in obviously reduced expression of ornithine decarboxylase 1 (ODC1), a recognized direct target of MYCN. ODC1 inhibition markedly undermines the promotive effects of CDC27 on NB cells in terms of proliferation, metastasis and the sphere-forming capacity (115).

### Ferroptosis-related compounds in neuroblastoma

There are currently several drugs for NB treatment, including cisplatin, etoposide, vincristine, doxorubicin, and cyclophosphamide. These drugs are the most effective inducers of apoptosis. However, this type of drug therapy creates multidrug-resistant clones, which makes eradicating this type of tumor much harder and favors tumor recurrence (116). The induction of ferroptosis through the use of drugs and agents in NB can be used to achieve better therapeutic outcomes, and this is also another hot topic in current research. Inducing ferroptosis has great potential as an anticancer therapeutic strategy in various NB tumor types, particularly in tumors with RAS mutations. The ferroptosis inducers erastin and RSL3 reduce RAS mutation-rich N2A cell (mouse neuroblastoma N2A cells) viability by increasing ROS levels and inducing cell death. In contrast, ferroptosis inhibitors lower the high ROS levels and reduce viability defects in erastin- or RSL3-treated cells. Ferritin (Fth) heavy chain 1, a ferrous oxidase that converts redox-active Fe2+ into redox-inactive Fe3+, may control the N2A-induced hypersensitivity response to ferroptosis. Overexpression of Fth reduces ROS levels and cell death and induces GPX4 expression. Additionally, NB cell lines present remarkably lower Fth expression than other cancer cell lines (117).

In addition, withaferin A (WA), a natural ferroptosis inducer in NB, activates Kelch-like ECH-associated protein 1 to activate the nuclear factor-like 2 pathway and produces increased intracellular unstable Fe(II) levels after heme oxygenase-1 is excessively activated, inducing ferroptosis or inactivating GPX4 (109). Chlorido[N,N’-disalicylidene-1,2-phenylenediamine]iron(III) complexes in NB cell lines produce lipid-based ROS and induce ferroptosis with greater efficacy than the therapeutic drug cisplatin (118).

## Conclusion

Despite advances in multimodal treatment, midbody treatment and the prognosis of gliomas and neuroblastoma are discouraging. Ferroptosis is a newly identified form of programmed cell death (PCD) dependent on iron that differs from apoptosis, cell necrosis, and autophagy. It plays a very important role in GBM and NB. This article summarizes the mechanisms involved in the roles of ferroptosis in GBM and NB. To summarize, we report that (1) the GPX4 pathway remarkably affects GBM and NB and that direct or indirect inhibition of GPX4 disrupts lipid peroxidation. (2) MYCN amplification may be a potent target in NB. (3) Nanodrugs may be new therapeutic agents for treating glioma and neuroblastoma. (4) The complexity of the tumor immune microenvironment and regulatory mechanisms need to be further explored.

Therefore, future research directions should include an in-depth study of ferroptosis, identification of key targets in the ferroptotic pathway and validation of their relationships in glioma and neuroblastoma, application of ferroptosis biomarkers in clinical prevention and monitoring, exploration of a new generation of ferroptosis-targeting systems, and finally, validation of the relationship between immunity and ferroptosis in glioma and neuroblastoma.

## Author contributions

HC and BL made equal contributions to the work. HX and GL took charge of project conception and design. HC and BL proposed the research and finalized the paper. QW, ZG and BF searched and summarized the literature. All authors contributed to the article and approved the submitted version.

## Glossary

CNS: Central nervous system
NB: Neuroblastoma
GBM: Glioblastoma
TMZ: Temozolomide
TTFields: Tumor-treating fields
ROS: Reactive oxygen species
GPX4: Glutathione peroxidase 4
FSP1: Ferroptosis suppressor protein 1
DHODH: Dihydroorotate dehydrogenase
NADPH: Nicotinamide adenine dinucleotide phosphate
GSH: Glutathione
SLC7A11: Recombinant solute carrier family 7, member 11
OTUB1: OTU domain, ubiquitin aldehyde binding protein 1
OTU: Ovarian tumor domain protease
NKAP: NF-κB activating protein
FXR1: Fragile X mental retardation syndrome-related protein 1
NKAP: NF-κB activating protein
RND1: Rho family GTPase 1
HD: , High cell density
ALDH1A3 Aldehyde dehydrogenase family 1: subfamily A3
ACSL4: Acyl-CoA synthetase long chain family 4
ALOX15: Arachidonate 15-lipoxygenase
PUFAs: Polyunsaturated fatty acids
PPARα: Peroxisome proliferator-activated receptor α
Hsp90: Heat shock protein 90
Drp1: Dynamin-related protein 1
PPAR: Peroxisome proliferator-activated receptor
BH4: Tetrahydrobiopterin
GCH1: GTP Cyclohydrolase1
NCOA4: Nuclear receptor coactivator 4
COPZ1: Coatomer protein complex subunit zeta 1
MXRA8: Matrix remodeling-associated protein 8
FTH1: Ferritin Heavy Chain 1
Nrf2: Nuclear factor erythroid 2-related factor 2
ATF4: Activating transcription factor 4
NOX4: NADPH oxidase 4
SOD1: Superoxide dismutase 1
PAB: Pseudolaric acid B
SOD1: Superoxide dismutase 1
ATG5: Autophagy related 5
VEGFR: Vascular endothelial growth factor receptor
NQO1: NAD(P)H quinone dehydrogenase 1
MDA: malondialdehyde
SDT: Sonodynamic therapy
BBB: Blood‒brain barrier
GIME: Glioma immunosuppressive microenvironment
TAM: Tumor-associated macrophages
ICIs: Immune checkpoint inhibitors
TILs: Tumor-infiltrating T cells
CAR-T: Chimeric antigen receptor T
DIPG: Diffuse intrinsic pontine glioma
AF: Amentoflavone
NOX: NADPH oxidase
PFI: Progression-free interval
LGG: Low-grade glioma
OS: Overall survival
Fpn: Ferroportin
FtMt: Mitochondrial ferritin
WA: Withaferin A
PCD: Programmed cell death
