# Supplementary material for: Opportunities and challenges related to ferroptosis in glioma and neuroblastoma
Source: Front Oncol. 2023 Mar 2;13:1065994. doi: 10.3389/fonc.2023.1065994 (PMC10021024; doi:10.3389/fonc.2023.1065994)
Supplement: Supplementary file 1 [file Table_1.pdf]

Supplement1 Ferroptosis-related gene signature in glioma

Databases	Genes	Application	Ref.
CGGA and TCGA	BAP1, GLS2, CISD1, PRNP, AKR1C3, TF, ACACA, ACSL6, MAP3K5, CDKN1A, G6PD, HSPB1, LOX, STEP3, ACSL1, CP, HMOX1, CYBB, ANO6, RB1, PCBP1, PGD, AURKA, G3BP1, and TP53	• The low-risk group had primary gliomas, LGG histology, low-grade, young group, IDH mutant type, 1p/19q codeletion, and MGMT promoter methylation.<br>• High-risk scores correlated with worse overall survival.	[93]
CGGA, TCGA and REMBRANDT	AKR1C3, AKR1C2, AKR1C1, NCOA4, EMC2, GCLC, CRYAB, FAD52, CBS, ZEB1, FDFT1, ACACA, HMGCR, SOLE, CS, SLC7A11, NFS1, ACSL3, CISD1, ACSL4, PEBP1, GOT1, GLS2, IREB2, TP53, RPLB, KEAP1, HSBP1, ACO1, CD44, NFE2L2, HSPB1, STEAP3, ABCC1, PGD, LPCAT3, SLC1AS5, CARS, GSS, TFRC, FANCD2, NOO1, GCLM, FTH1, ALOX5, SAT1, HMOX1, GEPD, PTGS2, MT1G, ACSF2, PHKG2, AIFM2, ATP5G3, GPX4, ALOX15, ALOX12, DPP4, CHAC1	• The high-risk score associated with FRGs predicted the glioma immunotherapy.<br>• The grade IV, IDH wild type and no deletion of 1p/19q had the high-risk score.<br>• The high-risk score associated with FRGs predicted the poor prognosis.	[94]
CGGA and TCGA	FTH1, SLC7A11, NOCA4, GOT1, TF, HSBP1, TFRC, SLC1A5, KIAA1429, FTL	• Higher risk scores had higher levels of immune checkpoint mRNA expression.	[95]
CGGA, TCGA and GTEx	ACSL3, CD44, HSPB1, PGD, STEAP3, CBS and FADS2	• Promoter DNA methylation might regulate the expression of FRGs in LGG with complexly regulative patterns.	[96]
CGGA and TCGA	IDH1, CD44, CAV1, DDIT4, TXNIP, VEGFA, RRM2, TP53, GLS2, GOT1, GABARAPL1, FBXW7, ENPP2, ATP6V1G2 and RGS	• Associated with microRNAs in cancer, central carbon metabolism in cancer, proteoglycans in cancer, 2-Oxocarboxylic acid metabolism and arginine biosynthesis.	[97]
CGGA and TCGA	ISCU、NFS1、MTOR、EIF2S1、HSPA5、AURKA、RPL8	• Predicted survival and treatment response in LGG.<br>• High-risk score was closely linked to characteristics of high malignancy in LGG.	[98]
TCGA, GEO (GSE4290 and GSE50161), GTEx, and CGGA	IDH1, CD44, CAV1, DDIT4, TXNIP, VEGFA, RRM2, TP53, GLS2, GOT1, GABARAPL1, FBXW7, ENPP2, ATP6V1G2 and RGS	• Identified novel ferroptosis-related genes with clinical value in glioma and revealed their possible tumor immune relevance.<br>• Pinpointed underlying critical elements of the chemokine, immune microenvironment and immune checkpoint.	[99]
TCGA, CGGA and GEO (GSEA16011 and GSEA61374)	GLS2、FDFT1、EMC2、DPP4、CS、CARS、RPL8、ATP5G3、ALOX15、ACSL4、TFRC、SLC1A5、SAT1、NCOA4, LPCAT3, FANCD2、CISD1 、CDKN1A、HSPB1、SLC7A11、NFE2L2、MT1G、HSPA5 and GPX4	• Highlighted the critical role of ferroptosis in TME formation and shaping.<br>• Quantitatively assessed ferroptosis levels in individual tumors to define the intratumor microenvironment and formulate precise treatment strategies for LGG patients.<br>• A higher score not only indicated resistance to TMZ therapy for LGG patients but also represented a poor prognosis in LGG patients either treated or not treated with TMZ.	[100]
TCGA and CGGA	ISCU、NFS1、MTOR、EIF2S1、HSPA5、AURKA、RPL8	• The risk score was found to be an independent prognostic factor.<br>• The model could effectively predict 12-, 36-, and 60-months OS and progression-free interval (PFI).<br>• More aggressive phenotypes presented in the high-risk group.<br>• Patients with higher risk scores may benefit from treatment with RTK pathway inhibitors.	[101]
TCGA, CGGA, and Rembrandt	DPP4, CAPG, HSPB1, AURKA, SESN2, PGD, ARNTL, LAMP2, EIF2AK4	• A total of three ferroptosis subtypes were identified by consensus clustering for prognostic FRGs.<br>• Perform a prognostic model which have the potential to screen the	[102]

		sensitivity to chemotherapy and immunotherapy in LGG patients.<br>• High-risk group is bound up with the process of tumor proliferation, is more sensitive to temozolomide therapy, and have a better response to anti-PD-1 therapy.	
TCGA, CGGA, and Gravendeel	CRNDE、LINC00844、FAM66C、TUBA3FP、SNHG8、HAR1A、LINC00641 and MYCNOS	• Constructed a risk signature by combining the risk signature and clinical parameters, which proved to be more accurate in predicting the prognosis of LGG.<br>• Differences in the levels of immune cell infiltration, immune-related functions, immune checkpoints, and m6A-related gene expression between the high- and low-risk groups.<br>• Higher levels of immune cell infiltration and more active immune-related functions in the high-risk group.	[103]
TCGA and CGGA	SAT1	• SAT1 expression was positively associated with TMB in LGG.<br>• The expression level of SAT1 was obviously correlated with the level of infiltrating macrophages and CD8 + T cells.<br>• The levels of most immune gene sets were associated with the SAT1 expression in LGG.<br>• OS and PFI of patients with LGG with high SAT1 levels were poorer than those with low SAT1 expression.<br>• SAT1 was closely associated with IDH mutation, 1p19q codeletion, chemoradiotherapy resistance and disease recurrence.	[104]
TCGA and CGGA	SPI, G6PD, ELVAL1, NNMT, ARNTL, CASP6	• Created a novel multigene signature to anticipate the prognosis and immunotherapy response of LGG patients.<br>• Patients in the high-risk subgroup had a poorer prognosis, greater immune abundance resided in the high-risk subgroup and increased ICGs expression existed in the high-risk subgroup.	[105]
